# Crowdsourcing to promote HIV testing among MSM in China: study protocol for a stepped wedge randomized controlled trial

**DOI:** 10.1186/s13063-017-2183-1

**Published:** 2017-10-02

**Authors:** SESH Study Group, Barry Bayus, Bolin Cao, Zihuang Chen, Maya Durvasula, Kevin Fenton, Rong Fu, David Glidden, Larry Han, Lisa Hightow-Weidman, Wenqi Hu, Shujie Huang, Michael Hudgens, Dianmin Kang, Haochu Li, Meizhen Liao, Chuncheng Liu, Wei Ma, Jessica Mao, Kate Mitchell, Katie Mollan, Jason Ong, Stephen Pan, Rosanna Peeling, Yilu Qin, Adam Saffer, Kumi Smith, Gabriella Stein, Songyuan Tang, Weiming Tang, Fern Terris-Prestholt, Joseph D. Tucker, Peter Vickerman, Cheng Wang, Chongyi Wei, Li Xue, Bin Yang, Ligang Yang, Wei Zhang, Tiange P. Zhang, Ye Zhang, Heping Zheng, Joseph D. Tucker

**Affiliations:** University of North Carolina Chapel Hill Project-China, No. 2 Lujing Road, Guangzhou, 510095 China

**Keywords:** HIV, HIV testing, Crowdsourcing, Men who have sex with men (MSM), stepped wedge randomized controlled trial, China

## Abstract

**Background:**

HIV testing for marginalized populations is critical to controlling the HIV epidemic. However, the HIV testing rate among men who have sex with men (MSM) in China remains low. Crowdsourcing, the process of shifting individual tasks to a group, has been increasingly adopted in public health programs and may be a useful tool for spurring innovation in HIV testing campaigns. We designed a multi-site study to develop a crowdsourced HIV test promotion campaign and evaluate its effectiveness against conventional campaigns among MSM in China.

**Methods:**

This study will use an adaptation of the stepped wedge, randomized controlled trial design. A total of eight major metropolitan cities in China will be randomized to sequentially initiate interventions at 3-month intervals. The intervention uses crowdsourcing at multiple steps to sustain crowd contribution. Approximately 1280 MSM, who are 16 years of age or over, live in the intervention city, have not been tested for HIV in the past 3 months, and are not living with HIV, will be recruited. Recruitment will take place through banner advertisements on a large gay dating app along with other social media platforms. Participants will complete one follow-up survey every 3 months for 12 months to evaluate their HIV testing uptake in the past 3 months and secondary outcomes including syphilis testing, sex without condoms, community engagement, testing stigma, and other related outcomes.

**Discussion:**

MSM HIV testing rates remain poor in China. Innovative methods to promote HIV testing are urgently needed. With a large-scale, stepped wedge, randomized controlled trial our study can improve understanding of crowdsourcing’s long-term effectiveness in public health campaigns, expand HIV testing coverage among a key population, and inform intervention design in related public health fields.

**Trial Registration:**

ClinicalTrials.gov, NCT02796963. Registered on 23 May 2016.

**Electronic supplementary material:**

The online version of this article (doi:10.1186/s13063-017-2183-1) contains supplementary material, which is available to authorized users.

## Background

Reaching marginalized populations with effective HIV prevention campaigns is critical to ending the HIV epidemic. Many public health campaigns promote healthy behaviors via social marketing, which is the systematic application of commercial marketing concepts to the planning, execution, analysis, and evaluation of programs [1, 2]. However, social marketing relies heavily on experts, limiting feedback from marginalized communities themselves [1–3]. Crowdsourcing may be a useful tool for engaging communities in public health interventions [4, 5]. Crowdsourcing comprises a large group of individuals solving a problem and then the solution is shared with the community. Crowdsourcing often involves open contests enabled through multi-sectoral partnerships [6, 7]. Originally developed in the private sector to improve products based on crowd inputs [7], crowdsourcing has been successfully used to advance health research [8]. For example, one contest generated a predictive model for breast cancer prognosis that outperformed previous approaches [9]. Another contest on amyotrophic lateral sclerosis (ALS) disease progression led to a winning clinical algorithm that was superior to ALS clinician assessments [10].

Crowdsourcing has several advantages over conventional approaches for the development of public health interventions. First, crowdsourcing is a bottom-up approach in which ideas stem from the community at large rather than experts at the top. This may promote creativity in the search for novel solutions. Second, by tapping into the collective wisdom of the community, crowdsourcing can increase community engagement and generate new messages that resonate among populations not typically reached by conventional approaches [3]. These diverse inputs may be particularly important to interventions that target marginalized populations who face multi-level barriers to care [11–13].

HIV testing is an essential service that fails to adequately reach many marginalized populations, including men who have sex with men (MSM) [14]. Global weighted estimates show that the rate of HIV testing in the past 12 months is only 31% among MSM in low-income and middle-income countries (LMICs) [15]. In China, the rapid spread of HIV in MSM led the government to significantly expand HIV control efforts [16]. However, several systematic reviews suggest that still only half of Chinese MSM have ever been tested for HIV [17–19]. Low levels of MSM community engagement, hesitancy to access facility-based services, and low trust in facility-based services all impede MSM HIV testing programs in China [20]. Current campaign efforts are not adequately reaching Chinese MSM, and new approaches are needed. A recent modeling study showed that a fourfold increase in general-population testing rates in China may prevent as many as 42,000 HIV infections and 11,000 deaths over the next 5 years [21].

There has been growing interest surrounding the use of crowdsourcing approaches to strengthen health interventions [3–5, 22]. Qualitative data from our group has shown that crowdsourcing contests empower individuals and result in a range of positive community engagement outcomes [23]. Furthermore, two preliminary studies conducted by our team suggest crowdsourcing may overcome challenges in expanding HIV services among MSM [22, 24]. In the first study, a crowdsourced HIV test promotion video was developed through an open contest, with its effectiveness evaluated against a conventional social marketing video. The study found that 37% of previously never-tested MSM who viewed the crowdsourced video subsequently reported receiving first-time HIV testing within the short term (4 weeks). This was similar to the testing rate observed in the group that viewed a social marketing video, but cost substantially less [22]. The second study evaluated the effectiveness of a crowdsourced condom promotion video against a social marketing video at 3 weeks and 3 months after the intervention. Results demonstrated the crowdsourced condom promotion video was non-inferior to the social marketing video and cost substantially less [24].

Despite crowdsourcing’s promise, the extent of crowd contribution to interventions remains limited. In addition, the effectiveness of crowdsourced interventions has not been examined in a range of local settings. Most crowdsourcing studies have been single contests that focused on generating campaign content, such as videos and posters, stopping short of designing an overall implementation plan. Our study aims to sustain crowd contribution through an entire intervention by implementing two serial contests - a content-focused contest followed by a second design-focused contest. Furthermore, our study will expand understanding of crowdsourcing’s effectiveness through a multi-site design that spans eight cities and assesses long-term effects. Results will reveal insights into qualities that are key to the success of public health interventions. The purpose of this article is to describe the design of a pragmatic stepped wedge randomized controlled trial aimed to develop and evaluate a crowdsourced intervention for promoting MSM HIV testing in China.

### Trial aims

Our study will develop an HIV testing intervention using crowdsourcing at multiple steps to sustain crowd contribution. The crowdsourced intervention will then be implemented and evaluated using a stepped wedge, randomized controlled trial (RCT) design. The control condition consists of conventional campaigns that are routinely conducted by local centers for disease control (CDCs) and community-based organizations (CBOs). Our first aim is to compare HIV test uptake associated with a crowdsourced intervention to that associated with conventional HIV test uptake campaigns. We hypothesize that a crowdsourced intervention is superior in eliciting HIV test uptake compared to conventional HIV test uptake campaigns. Our second aim is to compare secondary outcomes (including incremental cost, condom use, HIV-testing social norms, syphilis testing, etc.) in a crowdsourced intervention to those in conventional HIV test uptake campaigns. We hypothesize that a crowdsourced intervention is superior in promoting a range of healthy behaviors and HIV testing social norms.

## Methods/design

### Design

**Fig. 1:**
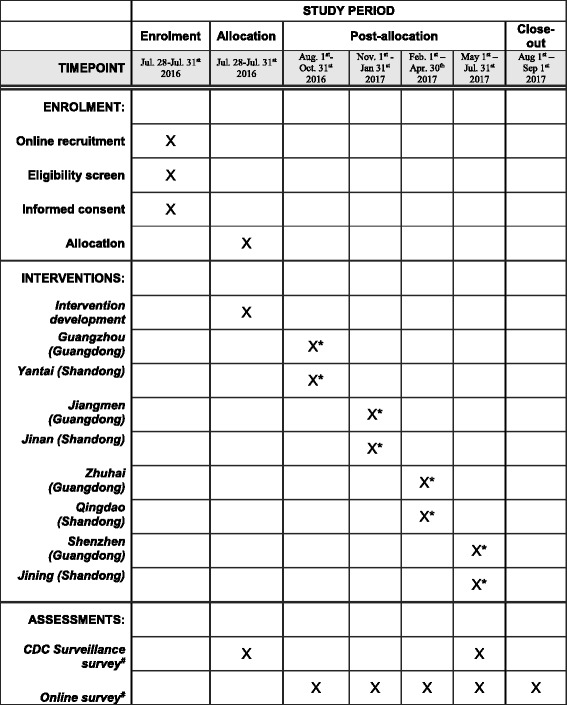
Stepped-wedge design of a crowdsourced intervention for promoting HIV testing in men who have sex with men (MSM) in China. Schedule of enrollment, interventions, and assessments. Time schedule of preparation and intervention phases are shown. Intervention development, online cohort recruitment, and baseline Chinese Center for Disease Control and Prevention (CDC) surveillance survey will take place during the preparation phase. In the intervention phase, a total of eight major metropolitan cities in China will be randomized to sequentially initiate interventions at 3-month intervals. A secondary CDC surveillance survey will take place during the last 3-month interval

This study will use an adaptation of the stepped wedge, RCT design. In the stepped-wedge RCT, study sites are randomized to begin the intervention at different times so that by the end of the study period all sites have initiated the intervention (Fig. 1). A total of eight major metropolitan cities - four from Guangdong Province (Guangzhou, Jiangmen, Zhuhai, Shenzhen) and four from Shandong Province (Yantai, Jinan, Qingdao, Jining) - will implement the crowdsourced intervention. These cities were chosen based on the following criteria: (1) previous CDC MSM sentinel surveillance site; (2) capacity for campaign implementation; and (3) capacity for intervention implementation at community level. Four cities (Guangzhou, Shenzhen in Guangdong Province, Qingdao, and Jinan in Shandong Province) will implement more intensive in-person events to promote engagement during the intervention development phase. Intervention development and implementation are overseen by our Social Entrepreneurship for Sexual Health (SESH) group and are detailed in later sections.

A number of factors influenced our decision to adopt a pragmatic stepped wedge, RCT design. Unlike a tightly controlled explanatory trial, a pragmatic trial evaluates an intervention in a real life context [25]. This aligns with our aim of examining whether crowdsourced interventions work in a range of local settings. A pragmatic design allows us to examine this intervention in eight different city clusters. The rationale for a stepped wedge cluster randomized trial allows the evaluation of the study intervention at a city-level rather than an individual level, and the random and sequential crossover of clusters from control to intervention ensures all clusters are exposed. The study design is appropriate given that the intervention draws on city-level media and programs, in addition to individual-level programs. In addition, previous studies demonstrate that crowdsourcing can enhance HIV interventions among MSM [22, 24, 26]. Given that we will recruit MSM, a key population with higher risk of acquiring HIV than the general population, withholding our intervention from a subgroup of participants would be difficult. A stepped wedge, RCT design addresses this concern by ensuring that all participants receive the intervention and by allowing each city to serve as its own control.

The eight cities are randomized to initiate intervention in groups of two at 3-month intervals (Fig. 1). The order of intervention implementation at four cities within each province (Guangdong and Shandong Provinces) was randomized by a researcher (WMT). He assigned each city a number; the intervention order was based on results from random number generation using the Mersenne-Twister pseudo-random number generator in SAS software. One city in Guangdong Province and one city in Shandong Province will then begin the intervention simultaneously, i.e. city-level randomization will be stratified by province.

While waiting to initiate the intervention, cities will be in the control condition. This consists of conventional testing campaigns that are part of the routine activities of the local CDC and CBOs. CDCs typically work with the local education department to develop educational material on HIV prevention. Educational materials are then distributed at CDC surveillance centers, where testing services are also available [27]. CBOs provide prevention-oriented outreach programs and some testing services to their target communities [28].

### Study setting and recruitment

We will establish an online cohort and build online survey tools using Sojump Survey Software (Sojump, Shanghai, China) (see Additional file 1 for online survey instrument). Men will enter the study through website and social media banner/word advertisements, though the survey-platform IP address restriction ensures that only those who live in the eight study cities can launch the questionnaire. China’s largest gay app, BlueD, will be used to target recruitment within the eight cities. Eligible men will be invited to join the online cohort. No names or addresses will be collected from participants. In addition to direct recruitment through websites and social media advertisements, participating individuals will be invited to refer up to three friends from their social networks within the eight cities and will receive a 10 RMB incentive for each successfully invited eligible participant. All individuals who enroll in the study will receive a 50 RMB (8.50 USD) prepaid cell phone card for the first follow up and 50 RMB for each subsequent follow up. Those who complete all surveys will be given an opportunity to win an iPad mini. Surveys will be given at baseline and every 3 months thereafter, with each participant completing a total of five surveys (Fig. 1). The allocation of the city clusters will occur before participants are recruited. After allocation, each local implementing team will be informed of the intervention date. Local implementation teams will coordinate intervention activities with relevant city organizations, though there will be no formal notification sent to MSM in the cohort, local providers, or others.

The MSM surveillance sites run by local CDCs in each of the eight cities will include additional questions about participant perceptions of SESH marketing images, social media engagement with SESH, contribution history in SESH contests, exposure to other ongoing campaigns, HIV/syphilis testing, and HIV/syphilis test results (see Additional file 2 for CDC surveillance survey instrument). Following informed consent, cell phone numbers will be used to link CDC and online survey datasets. The SESH research group will manage and oversee data collection.

### Eligibility criteria for men participating in the intervention

Eligibility criteria will include: currently living and planning to live in the eight cities for the next 12 months; not living with HIV; no HIV testing in the past 3 months; born biologically male and identifying as either male or transgender; had anal sex with men at least once during their lifetime; age 16 years and older; willing to provide cell phone number (for follow up and incentive delivery purposes); and having completed the informed consent document. The consent document for participation in the trial was viewed online by potential participants and electronically signed by those willing to participate in the trial. All participants were asked by the researchers to provide consent to take part in the study. Taking part in the study involved completing a survey about behaviors every 3 months. MSM who meet all other eligibility criteria but who were tested for HIV within the past 3 months or are living with HIV will be invited to complete a single survey, but will not be followed over time in the cohort.

### Intervention

#### Part 1 - intervention development

**Fig. 2:**
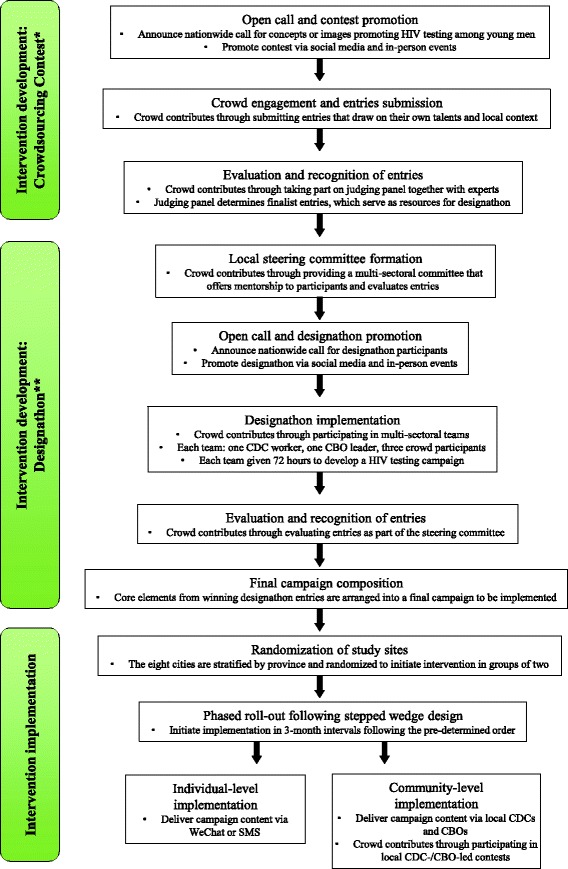
Schematic of crowdsourcing intervention development and implementation. The block diagrams describe the steps for intervention development and implementation. The text also highlights how crowd contribution will be sustained. Intervention development consists of the crowdsourcing contest followed by the *designathon*. Intervention implementation will take place at the individual level and community level, with crowd feedback from the designathon. *Crowdsourcing contest solicits concepts or images for promoting HIV testing and produces winning entries selected by the crowd and expert judges. **Designathon adopts the concept of a *hackathon* and allows diverse individuals to intensely collaborate on designing a comprehensive HIV test promotion campaign. CDC Chinese Center for Disease Control and Prevention, CBO community-based organization, SMS short message service text

The intervention will be developed from a nationwide crowdsourcing contest and a *designathon* (Fig. 2). The crowdsourcing contest will generate intervention materials that will later be packaged during the designathon into core elements of an HIV testing campaign. This ensures crowd wisdom is utilized through the entire intervention, from idea generation to campaign implementation. Exact details of the intervention are contingent on the outcomes of the crowdsourcing contest and the designathon.

##### Crowdsourcing contest

The first part of the crowdsourcing contest will be an open call for concepts (<500 characters) or images (photographs, posters, drawings, etc.) promoting HIV testing among young men in China. This open call will be announced on social media platforms nationwide. Social media promotion will include QQ, Weibo, and WeChat announcements, and short videos explaining the contest from SESH and our community partners in each city (CBOs and student groups interested in HIV testing). Social media will also serve as a channel for announcing prizes, deadlines, and other relevant information. Four cities (Guangzhou, Shenzhen in Guangdong Province; Qingdao, Jinan in Shandong Province) will implement in-person events in addition to social media promotion. In-person events will include community-based introductions, interactive feedback sessions, and community-driven events (decided by community partners). Multiple incentives, including chances to win an iPad Mini, cash, post cards, etc., will be included to encourage contest participation.

Crowdsourced entries will be evaluated by a crowd panel and an expert panel. The crowd panel will consist of MSM from each of the eight cities while the expert panel will consist of professionals from the local CDC, CBOs, and universities in the eight cities. These local panels increase the likelihood that local preferences will be incorporated into judging decisions, which may facilitate later implementation. The quality of crowdsourced ideas will be judged based on four established dimensions: novelty, relevance, feasibility, and elaboration. Judges will consider the four dimensions and score an entry on a 10-point single-item scale. Given that having a large number of judges evaluating a relatively small number of entries has been shown to be an internally consistent and externally valid approach [29], each judge in our contest will evaluate no more than 20 entries. Based on the number of entries, we will ensure enough judges are recruited so that each entry has at least three independent ratings.

Following these judging criteria, all entries will first be screened to check for relevance to our contest and plagiarism. Next, the crowd panel and the expert panel will score the entries. The top 40 concepts and/or images will be selected as finalist entries and be presented as materials for the designathon. Following the judging process, contributions will be recognized with prizes and acknowledgement. The first three places from the expert panel judging and the first place from the crowd panel judging will be recognized with prizes. All other entries will be recognized with a participation certificate. The crowdsourcing contest and judging are planned to span a 3-month period.

##### Designathon

The designathon will utilize finalist concepts and/or images to develop core elements of an HIV testing campaign, which includes the campaign content and the implementation plan. A designathon is similar to a hackathon, [30, 31] but focuses instead on designing a campaign. Teams are formed with an emphasis on multi-sectoral partnership. Each team consists of one CDC worker and one MSM CBO leader from each of the eight cities and three participants selected from a nationwide application. Teams will have 72 hours to brainstorm and generate a written intervention plan that incorporates new ideas and concepts from the crowdsourcing contest.

A steering committee will be formed with local professionals in public health, communications, civil society, and design. This steering committee will be responsible for providing feedback to teams during the designathon and judging the intervention plans that result from the designathon. Following the judging process, the first-place team will be recognized with a cash prize while other teams will be recognized with participation certificates. The design elements of the winning entry will be included in a final HIV test promotion campaign to be evaluated through the stepped-wedge RCT.

The final campaign will be implemented at both the individual and community levels using social media, in-person events, and other crowdsourced ideas for implementation. After the RCT is complete, we will launch an image bank that allows free access to images/taglines/concepts developed as part of the contest.

#### Part 2 - intervention implementation

Phased implementation will be carried out in the eight cities following the stepped wedge, RCT design (Fig. 1). Different from the traditional randomized trials, all the participants in this stepped-wedge RCT will receive the intervention, although the intervention initiation time will be different in each city. Thus, neither the participants, the care providers, outcome assessors, nor the data analysts will be blinded after assignment to interventions. Two cities in each step will sequentially receive the intervention for 3 months over a 12-month period (i.e. total of four steps). No transition period will be needed to embed the intervention as the predesigned interventions immediately start and finish. The rapid turnaround is possible because of social media components and pre-trial planning. All participants from the eight cities are recruited at baseline and will receive an online survey at baseline and every 3 months thereafter at the same calendar time, for a total of five surveys per participant. The online survey allows us to recruit enough participants in a short period (within 3 days). Implementation in each city will be locally adapted based on crowd feedback from the contest and the designathon. The intervention will be implemented at the individual level (via WeChat messages and SMS text messages) and at the community level (via community partners including CDC, CBOs, and social media influencers) (Fig. 2).

For individual-level implementation, the campaign content from the designathon will first be shown to the online cohort at the end of the baseline survey, and then repeated once every 2 weeks in the 3-month intervention interval. Half of the online cohort will receive the campaign content via WeChat message while the other half will receive the campaign content via SMS text message. For community-level implementation, community partners in each city will facilitate the campaign using crowdsourced implementation ideas generated from the designathon. The SESH research team will promote participant retention and follow-up completion using social media platforms.

### Study measures and outcomes

Information on socio-demographics, sexual behaviors, and psychosocial conditions will be collected using standardized online survey tools. Socio-demographic characteristics include participants’ age, highest level of education completed, annual income, marital status, sexual orientation, and sexual orientation disclosure. Behavioral and psychosocial variables include self-reported HIV testing, syphilis testing, HIV self-testing, HIV test-associated stigma, frequency of sex, and condom use (sex without a condom, sex always with condom, or no sex), HIV-testing social norms, HIV testing self-efficacy, community engagement, campaign engagement, and MSM empowerment.

The primary outcome of this study will be HIV test uptake in the past 3 months, assessed by self-reports during follow-up surveys and triangulated with HIV testing rates from CDC surveillance data during the same period. An increase of 10% in testing rate (assuming a proportion of HIV testing of 35% during the crowdsourced intervention period and 25% during the conventional intervention period) was chosen as the superiority margin. This choice was based on existing levels of HIV testing and judgments about feasible, important public health outcomes in the Chinese context.

A number of secondary outcomes will also be measured. These include syphilis testing, sex without a condom, community engagement, testing stigma, and others (see Additional file 3 for table of secondary outcomes). Outcomes will also be stratified based on a participant’s level of engagement during intervention development and based on their personal level of engagement during the stage of intervention implementation.

### Timeline

The study will span approximately 16 months. The first 3 months will be the preparation phase. A crowdsourcing contest followed by a designathon will be promoted and held to generate a crowdsourced campaign. CDC surveillance surveys will be conducted in the eight target cities. All interventions will be fully developed before the trial begins. The following 12 months will be the intervention phase. The intervention will be sequentially rolled out in each of the eight cities following the stepped-wedge design outlined in Fig. 1. The online cohort will be surveyed at baseline and every 3 months thereafter. A secondary CDC surveillance survey will also take place during the last 3-month interval. By the 16^th^ month, all cities will have implemented the intervention for a 3-month interval and the final follow-up survey will be conducted. The SESH research group will manage and oversee intervention progression.

### Sample size

We used a binary outcome, stepped wedge, RCT design for sample-size calculation. The required sample size is calculated for the primary outcome (see Additional file 4 for sample-size calculation table). To calculate sample size, we assumed that a crowdsourced intervention will be superior to a conventional method in promoting HIV testing among MSM who have not tested in the past 3 months. No cluster variation is expected. Assuming a proportion of HIV testing of 35% during the crowdsourced period and 25% during the conventional period, a total of eight clusters, four total intervention time periods, a coefficient of variation of 0.4 (usually between 0.15 and 0.4), two-sided alpha = 0.05, 90% power, and 30% loss to follow up, the total sample size is 1040 men (130 for each city). To further improve the power for sub-analysis and secondary outcomes, we increased the sample size to 1280 men (160 for each city). The calculation was made using the formulas developed by Michael A. Hussey et al. [32] (http://faculty.washington.edu/jphughes/pubs.html).

### Data management

All data from baseline and follow-up surveys are entered directly into computers and transmitted securely using SSL (TLS) 128-bit encryption. Data will be located in a dedicated server at UNC Chapel Hill. Data can be readily downloaded and converted to the format of commercially available statistical software. Survey responses will be kept separately from participants’ email addresses; the two files will be linked with a non-descript identifier that is encrypted and password-protected. An independent external advisory committee consisting of STI experts has been formed. The committee will meet periodically to review and evaluate data collection and study progress.

### Analysis

The primary outcome will be self-reported HIV testing uptake in the past 3 months, evaluated at the end line. We will examine a hypothesis comparing the superiority of the crowdsourced intervention with conventional HIV-test uptake campaigns. In our study, since the outcome is binary, generalized linear mixed models (GLMM) and generalized estimating equations (GEE) can be used for the primary outcome analysis. However, since we only have eight clusters, GLMM will be used for primary data analysis, as GLMM is preferred in studies with a small number of clusters [33]. The model will include intervention status and time as fixed effects and site and individuals as random effects. The estimated intervention effects will be reported with 95% CIs and *p* values. Descriptive analysis will be used to summarize the characteristics and behaviors of the participants at baseline and in the follow-up surveys.

Similar analyses will be conducted for binary secondary outcomes (continuous variables will be categorized into binary variables), including frequency of syphilis testing, frequency of HIV testing (among those with previous HIV testing), se without condoms, community engagement, awareness of HIV status, empowerment, and others. In addition, since four cities will implement more intensive in-person events to promote engagement (Guangzhou, Shenzhen, Qingdao, and Jinan) during intervention development, sub-analysis will be conducted to evaluate the potential effect of in-person events to promote HIV testing and other secondary outcomes among Chinese MSM. In addition, secondary analysis will investigate an interaction effect between intervention and community engagement, both for engagement during the intervention development stage and engagement during the intervention implementation stage at a personal level.

Sub-analyses will include: comparison of the effects of the intervention in participants with different ages (under 30 vs. 30 years or older), comparison of the effectiveness of two delivery methods for individual-level intervention (WeChat message vs. SMS text message), and comparison of the intensity of exposure to the intervention within one method (individual text messages/WeChat messages) and between methods (individual-level vs. community-level intervention).

Our team will distribute the results of this research to local, regional, and national stakeholders. We will not use any professional writers in writing the main manuscript and all decisions about authorship will follow conventions established by the International Committee of Medical Journal Editors. We plan to make all crowd-generated images, concepts, and related materials widely available under creative commons attribution. The main manuscript will include a full protocol, participant-level dataset, and statistical code.

## Discussion

HIV testing rate remains low despite persistent campaign efforts among MSM in China [17]. Novel strategies are urgently needed to promote HIV testing and strengthen public health interventions. Crowdsourcing has shown potential to enhance HIV testing and condom use among MSM in preliminary studies [22, 24]. Crowdsourcing can be a powerful vehicle for enhancing open innovation, building community engagement, and increasing the diversity of inputs in public health intervention. These qualities may be crucial to improving HIV services for MSM, who face multi-level barriers that require inputs from a range of disciplines [11–13]. We believe a large-scale, multi-site stepped-wedge RCT is needed to evaluate crowdsourcing’s ability to be effective in the complex context of local settings.

Several limitations should be considered when conducting crowdsourced interventions. First, the use of social media for contest promotion and data collection may overlook individuals who lack access to online tools. The incorporation of in-person events held by CBOs and data collection by MSM surveillance sites may mitigate this issue. Second, crowd judging is prone to bias, inconsistent judging criteria, and reliance on popular opinion. The inclusion of experts, both in the crowdsourcing contest’s judging panel and the designathon’s steering committee, may offset these effects by balancing crowd preferences with professional inputs. Third, all behavioral measures will be self-reported, increasing the likelihood of social desirability bias. Further, there may be a potential for the Hawthorne effect: men may report a change in behavior due to their awareness of participating in the trial. However, our online computer-based survey allows a high degree of anonymity that can reduce the strength of this bias. Furthermore, triangulation with CDC surveillance site data on HIV testing can facilitate validation. Fourth, we will treat time as a fixed variable, which may be a biased estimate. We will conduct a sensitivity analysis and treat time as a random effect to account for secular trends or risk-biased estimates of the effect [33]. We will also conduct a sensitivity analysis that uses randomization inference to test for the effect [34].

Our study will generate important research and policy implications regarding the use of crowdsourcing methods in public health. The study outcomes will help guide policy and intervention practice of governmental departments and community-based organizations regarding the expansion of key population HIV testing strategies. Moreover, practical knowledge gained from developing and implementing a crowdsourced intervention may be applicable for future efforts to scale-up HIV testing. If successful, this model of crowdsourcing-based intervention development and implementation can be applied toward improving other major public health services.

### Trial status

At the time of this draft, intervention development and participant recruitment have begun. Study outcomes, data cleaning, and analysis are pending. The study is registered in the Clinical Trials.gov database (NCT02796963). The database will also be used for documenting protocol modifications. The trial protocol conforms to the Standard Protocol Items: Recommendation for Interventional Trials (SPIRIT) 2013 statement (see Additional file 5, Fig. 1) [35].

## Additional files

Additional file 1: Online survey instrument (English version). This is the English version of the baseline online survey for our online cohort. (DOCX 95 kb)

Additional file 2: CDC surveillance survey instrument (English version). This is the English version of the CDC surveillance survey baseline online used at CDC surveillance sites. (DOCX 46 kb)

Additional file 3: Table for secondary outcomes. This table lists the secondary outcomes measured in this RCT and their definitions. (DOCX 35 kb)

Additional file 4: Table for sample size calculation. This table lists the variables and values involved in the sample size calculation. (DOCX 46 kb)

Additional file 5: Spirit 2013 checklist. This document lists the recommended items to address in a clinical trial protocol and related documents. (DOC 123 kb)

## Abbreviations

CBO: Community-based organization
CDC: Chinese Center for Disease Control and Prevention
CI: Confidence interval
GD: Guangdong Province
GEE: Generalized estimating equations
GLMM: Generalized linear mixed models
HIVST: HIV self-testing
LMIC: Low-income and middle-income countries
MSM: Men who have sex with men
RCT: Randomized controlled trial
SD: Shandong Province
SESH: Social Entrepreneurship for Sexual Health Group
SMS: Short message service text message
SSL: Secure sockets layer
TLS: Transport layer security
