# Correction to Crowdsourcing to promote HIV testing among MSM in China: study protocol for a stepped wedge randomized controlled trial

**DOI:** 10.1186/s13063-017-2258-z

**Published:** 2017-10-23

**Authors:** SESH Study Group, Joseph D. Tucker

**Affiliations:** University of North Carolina Chapel Hill Project-China, No. 2 Lujing Road, Guangzhou, 510095 China

## Correction

In the original publication [1] are errors in the acknowledgement section in the backmatter. The corrections can be found below in this Erratum.

Incorrect affiliation 4:

Guangdong Provincial Center for Skin Diseases and Sexually Transmitted Infections Control, No. 2 Lujing Road, Guangzhou, China, 510095.

Correct affiliation 4:

Guangdong Provincial Center for Skin Diseases and Sexually Transmitted Infections Control Center, Dermatology Hospital, Southern Medical University, No. 2 Lujing Road, Guangzhou, China, 510095.

Incorrect author affiliation:

Meizhen Liao^15^, Dianmin Kang^15^

Correct author affiliation:

Meizhen Liao^16^, Dianmin Kang^16^

Missing affiliation 16:

Shandong Provincial Center for Disease Control and Prevention, Jinan, China
