# Supplementary material for: Crowdsourcing to promote HIV testing among MSM in China: study protocol for a stepped wedge randomized controlled trial
Source: Trials. 2017 Oct 2;18:447. doi: 10.1186/s13063-017-2183-1 (PMC5625620; doi:10.1186/s13063-017-2183-1)
Supplement: Supplementary file 1 — Online survey instrument (English version). This is the English version of the baseline online survey for our online cohort. (DOCX 95 kb) [file 13063_2017_2183_MOESM1_ESM.docx]

**Additional File 1. Online survey instrument (English version)**

**Men’s Health Study**

**About this Study:**

You are being asked to take part in a research study that will help us better understand sexual behavior and HIV testing among men in China. Your participation in this project will allow us to develop better interventions to improve sexual health among men across China.

**What’s involved?**

Our study participants consist of men from eight cities including Guangzhou, Shenzhen, Zhuhai, Jiangmen, Jinan, Qingdao, Yantai, and Jining. We will use your IP address to determine whether you live in one of these eight cities. If you participate in this study, you will be asked to complete an online questionnaire. A subset of participants will be asked to complete four additional follow-up questionnaires. The questionnaires will ask you to provide sociodemographic information and information about your sexual behaviors. In order to ensure that your privacy is protected, all of your online responses will be encrypted and securely transferred to our data servers.

Upon completion of this survey, you will receive 50 RMB. We will ask eligible participants to complete one follow up questionnaire every three months for one year. Each time you complete a follow up questionnaire, you will receive 50 RMB. If you complete all four questionnaires, you will have the chance to win an iPad mini.

All research on human volunteers is reviewed by a committee that works to protect your rights and welfare. If you have questions or concerns, or if you would like to obtain information or offer input, please contact the UNC Institutional Review Board by email at [IRB_subjects@unc.edu](mailto:IRB_subjects@unc.edu). Our IRB code is 16-0851. If you have any questions about the research or your participation in the study, feel free to contact Rong Fu (Guangzhou, 18613067997) or Haochu Li (Jinan, 15169132257).

**Q. Basic Information (Eligibility Survey) (Q1-5)**

Q1. Do you live in one of the following cities, and do not have moving plans in the next 1 year?

- Guangzhou
- Shenzhen
- Zhuhai
- Jiangmen
- Jinan
- Qingdao
- Yantai
- Jining
- None of above (Not eligible to take this survey – Skip to End of Survey)

Q2. How would you describe your assigned sex at birth?

- Male
- Female (Not eligible to take this survey – Skip to End of Survey)

Q3. Please enter your age:

____years-old (Not eligible to take this survey if the year is greater than Launch day + 1999 or < 16 y/o – Skip to End of Survey)

Q4. In your lifetime, have you ever had anal sex with another man?

- Yes
- No (Not eligible to take this survey – Skip to End of Survey)

Q5. Will you agree to participate in our follow-up surveys lasting for the next year, occurring once every three months? For every follow-up survey you complete, you will receive a 50 RMB reward.

- Agree
- Decline (Not eligible to take this survey – Skip to End of Survey)

Q6. Will you agree to provide us your Chinese mobile phone number? (Answering this question is required to participate in the survey and to receive your reward for participating. We will not distribute your number to any agency or individual. Thank you for your cooperation.)

- Agree
- Decline (Not eligible to take this survey – Skip to End of Survey)

Q7. Please enter your mobile number:

**Online Consent Form**

**Title of Study:** Men’s Health Study

**IRB study number:** 16-0851 **Principal Investigator:** Dr. Joseph Tucker
Dr. Joseph D. Tucker, UNC Project-China, Number 2 Lujing Road, Guangzhou, China,

**What are some general things you should know about this research studies?** You are being asked to participate in a research study. To join this research study is voluntary. You may for whatever reason refuse to join or withdraw your consent to be in the study at any time, without penalty. Details about this study are discussed below. It is important that you understand this information so that you can make an informed choice about joining this research study.

**What is the purpose of this study?** Innovative approaches to HIV testing promotion campaigns are urgently needed. The current strategy to developing many of these campaigns is to repackage old ideas rather than create new ones. The purpose of this research study is to understand how crowdsourcing can be used to leverage both the high Internet use and willingness to participate in online forums of young MSM (men who have sex with men) to transform the design and implementation of HIV testing promotion campaigns. Crowdsourcing is the process of taking a task traditionally performed by a single individual or organization, and instead outsourcing the task to a large group to complete in the form of a contest or open call, often enabled by the Internet.

**How many people will take part in this study?** If you decide to participate in this research study, you will be one of approximately 1700 individuals recruited across eight Chinese cities including Guangzhou, Shenzhen, Zhuhai, Jiangmen, Jinan, Qingdao, Yantai, and Jining.

**What will happen if you take part in the study?** Your part in this current survey will last approximately 20-30 minutes. Follow-up surveys will last approximately 10-15 minutes. During this current survey, you will be asked to complete an online questionnaire. Upon completion of this initial questionnaire, you will be asked to input your mobile phone number as a means for the research team to prevent duplicate responses, to send reminders, and to distribute rewards for participation. A subset of participants will be asked to complete one follow up survey once every three months for a year, consisting of four follow up surveys in total. If you do not respond to the initial follow up request, you will receive a message reminder. To do this, we will also ask you to provide your WeChat number. The study questionnaires will ask you to provide sociodemographic information as well as details about your sexual health and sexual activity.

**What are the possible benefits from being in this study?** Research is designed to benefit society by gaining new knowledge. The proposed study will make important contributions to the sexual health literature. The field of HIV interventions among young MSM in resource-limited settings is in its infancy. The results from this study will help the research team develop a MSM targeted, community-level intervention that will be fielded and evaluated in the Chinese setting. Your participation will also help design better interventions to promote HIV testing among MSM in China.

**What are the possible risks or discomforts involved from being in this study?** We will ask participants to provide sensitive information about their sexual partners and practices. Participants may feel embarrassed, anxious, or otherwise distressed by providing information of such a personal nature. Participants may also experience fatigue in response to the proposed evaluations (e.g. from looking at a computer screen). Some participants might fear that refusal to participate in the study might jeopardize their sexual orientation identity – especially if the participant has not come “out” to him or herself and/or the community). Other participants may fear that the research staff might “out” them or discuss their private details with other (MSM and non-MSM) members in their community. While the risk is minimal, there is still the possibility for breaches of confidentiality.

**How will your privacy be protected?** All data is directly entered into computers as participants complete the questionnaires. Programs to ensure accuracy, completeness, and internal consistency are automated. Data can be readily downloaded and converted to the format of commercially available statistical software. During collection of the online portion of the study, all data will be transmitted securely using SSL (TLS) 128 bit encryption across the Internet (HTTP). SSL provides users with the assurance of access to a valid, “non-spoofed” site, and prevents data interception or tampering with sensitive information. The SSL certificate that will be used for this project will use 128-bit encryption, the preferred security level of government and financial institutions. 128-bit encryption offers protection that is virtually unbreakable. For example, if a hacker could crack a standard 40-bit SSL session in a day, it is estimated that it would take well beyond a trillion years to accomplish the same thing against a 128-bit SSL session. A dedicated server, which eliminates security issues involved with shared hosting environments where hundreds of websites and users reside on one shared web server as well as ensuring both physical and network security, will be used to house the data. Data will be located in a secured server at UNC Chapel Hill.

The server will be configured with redundant hard drive array to ensure reliability. Access to the data will be password protected within the server’s firewall. Survey responses will be kept separately from participants’ email addresses; the two files will be linked with a non-descript, unique, randomly generated identifier. Only the PI and a designated senior staff member will have the password to access to the “key” that links the nondescript identifier to personally identifiable information. Cookies will not be used in any way to track participant activity.

**What if you want to stop before your part in the study is complete? If at any point in the study you do not want to answer a question or no longer want to participate,** you can stop and withdraw from this study without penalty. The investigators also have the right to stop your participation if you have an unexpected reaction, have failed to follow instructions, etc.

**Will you receive anything for being in this study? Will it cost anything? Participants will have the opportunity to earn up to 250 RMB credit on their mobile phone or wechat red pocket – this credit will be distributed as 5 separate 50 RMB mobile phone recharges/wechat red pocket top-ups-and an iPad mini. Participants will receive 50 RMB upon completion of the first questionnaire, and 200 RMB for the 3/6/9/12 month follow up surveys if they are eligible. For those who complete all of the follow up surveys, they will have a chance to win an iPad mini.** There are no costs associated with participating in this research study.

**What if you have questions about this study?** If you have any questions, complaints, or concerns about the research or your participation in the study, feel free to contact Rong Fu (Guangzhou, 18613067997) or Haochu Li (Jinan, 15169132257)

**What if you have questions about your rights as a research participant?** All research on human volunteers is reviewed by a committee that works to protect your rights and welfare. If you have questions or concerns, or if you would like to obtain information or offer input, please contact the Institutional Review Board at 020-87255824 or send an email to [IRB_subjects@unc.edu](mailto:IRB_subjects@unc.edu). You may also contact the Guangdong Provincial Skin Diseases & STI Control Center IRB at 020－83027652 or by email to [sesh@seshglobal.org](mailto:sesh@seshglobal.org).

Q8. If you understand and agree to participate in this research study, please select “Agree” from the options below. We thank you for your participation!

- Agree
- Decline (Skip to End of Survey)

**Survey Access**

Q9. How did you find out about our research study?

- Blued’s banner ad
- Danlan webpage banner ad (www.danlan.org)
- Weibo banner ad
- Weixin banner ad
- QQ group
- Friend referral
- Other

Q10. What device are you using to access our research study?

- Desktop or laptop computer
- Mobile phone
- Tablet device

**A. Sociodemographics**

*The next set of questions will ask you to provide some information about yourself.*

A1. Which kind of hukou are you currently holding?

- Current city’s hukou
- Other urban hukou in the province
- Rural hukou in the province
- Urban hukou outside the province
- Rural hukou outside the province

A2. How long have you lived in this city? ______years_________months (Note: this refers to the duration, not the specific year and month)

A3. What is your current living situation?

- Live in a house or apartment that I own
- Live in a house or an apartment I rent
- Rent a room or space in someone else's house or apartment
- Stay with someone else for free
- Live at my workplace (on-site shelter, dorm, etc.)
- Live on the street, homeless, or stay at more than two different places a week
- Other

A4. What’s your occupation?

- Student
- Civil servant
- Farmer
- Labor worker (blue collar)
- Office worker (white collar)
- Seller/service staff
- Technician
- Sex worker
- Unemployed
- Other______

A5. What is your current legal marital status (referring to women)?

- Not married
- Engaged or Married
- Separated or Divorced
- Widowed

A6. Do you have any child now?

- Yes
- No

A7. What is the highest level of education that you have **completed**?

- High school or below (including Zhongzhuan)
- Some college (Dazhuan)
- College/Bachelors
- Masters/PhD or above

A8. What is your total individual **monthly** income from all sources?

- Less than 1500 RMB
- Between 1500 and 3000 RMB
- Between 3001 and 5000 RMB
- Between 5001 and 8000 RMB
- Greater than 8000 RMB

A9. What is your gender identity?

- Male
- Female
- Transgender
- Unsure/Other

A10. What is your sexual orientation?

- Homosexual
- Bisexual
- Heterosexual
- Unsure/Other

A11. Have you ever told anyone about your sexuality or sexual history with men (except your sexual partner)?

- Yes
- No (Skip to Section B)

A12. Have you ever told health-care providers about your sexuality or sexual history with men?

- Yes
- No

**B. MSM Basic Situation**

*The next set of questions will ask you about your sexual behaviors with other men.*

**Section 1: General information**

B1. How old were you when you had anal intercourse with another man for the first time?

________years old *(Number input)*

B2. The first time you had sex with a man, was it consensual?

- Yes
- No
- *Our definition of consensual is that you were interested in having sex with him, but that you felt like you could have refused or stopped it if you wanted to.*

B3. In the last 12 months, where did you mainly meet with your male sexual partners? (Select all that apply)

- Pub, disco, tearoom, or club
- Spa or bath house, sauna, foot or body massage parlor
- Park, public restroom, public lawn
- Website
- Social media (Blued, Jackd, QQ, etc)
- Through friends
- Other
- I didn’t meet new sexual partner

B4. When did you last have anal sex with a male partner?

- Within the last 3 months
- More than 3 months ago (skip to B15)

B5. In the last three months, approximately how many different male sexual partners did you have anal sex with?

_________male sex partners *(Number input) (Must be >=1)*

B6. In the last three months, when you had anal sex with your male sex partner, how frequently did you use condoms?

- 0% condom use
- Less than 50% condom use
- More than 50% condom use
- 100% condom use

B7. In your last sexual intercourse with any male partner, did you or your sex partner use condoms?

- Yes
- No

**Section 2: Stable male sex partners**

*Stable male partners mean male sex partners who maintain a sexual relationship with you more than 3 months (﹥3 months), including boyfriends and/or regular male sex partners.*

B8. In the past three months, have you had any stable male sex partners?

- Yes
- No (skip to B15)

B9. In the past three months, how many stable male sex partners have you had?

_______________ (Number input)

B10. Where did you meet your stable male sex partners? (Choose all that apply)

- Pub, disco, tearoom, or club
- Spa or bath house, sauna, foot or body massage parlor
- Park, public restroom, public lawn
- Website
- Social media (Blued, Jackd, QQ, etc)
- Through friends
- Other

B11. In the last three months, have you had anal sex with your stable sex partner(s)?

- Yes
- No (skip to B15)

B12. In the last three months, when you had anal sex with your stable partner(s), what role did you assume?

- Always insertive (always 1)
- Mostly insertive (mostly 1)
- Both insertive and receptive in similar amounts (Both 1 and 0 in similar amounts)
- Mostly receptive (mostly 0)
- Always receptive (always 0)

B13. In the last month, how many times have you had anal sex with your stable male sex partners in total? ___________(Number input)

B14. Out of the anal sex you had with your stable male sex partners in the last month, how many times was a condom used? ___________(Number input)

B15. What’s the HIV status of your most recent stable partner?

- HIV positive
- HIV negative
- No idea (skip to B17)
- I never had a stable partner (skip to Section 3)

B16. How did you know his (the most recent stable partner) HIV status?

- He told me some time ago / I had known for some time
- He told me (online or in person) before sex
- I knew it from his profile on the Internet
- He made it clear without actually telling me
- Someone else told me
- We were at an event where everyone was HIV negative
- We were at an event where everyone was HIV positive
- I guessed
- Other reason

B17. Please count the number of your stable partners(s) with different HIV statuses in your lifetime:

- HIV positive ___________(number input)
- HIV negative ___________(number input)
- HIV unknown __________(number input)

**Section 3. Casual male sex partners**

*Casual male sex partners means male sex partners who maintain a sexual relationship with you equal to or less than 3 months, including commercial sex partners (≤3 months).*

B18. In the past three months, have you had casual male sex partners?

- Yes
- No (skip to B25)

B19. In the past three months, how many casual male sex partners have you had?

_______________ (Number input)

B20. Where did you meet your casual male sex partners?

- Pub, disco, tearoom, or club
- Spa or bath house, sauna, foot or body massage parlor
- Park, public restroom, public lawn
- Website
- Social media (blued, jackd, QQ, etc)
- Through friends
- Other

B21. In the last three months, have you had anal sex with your casual sex partner(s)?

- Yes
- No (skip to B25)

B22. In the last three months, when you had anal sex with your casual male sex partners, what role did you assume?

- Always insertive (always 1)
- Mostly insertive (mostly 1)
- Both insertive and receptive in similar amounts (Both 1 and 0 in similar amounts)
- Mostly receptive (mostly 0)
- Always receptive (always 0)

B23. In the last month, how many times have you had anal sex with your casual male sex partners in total? (Include all casual male sex partners) ___________(Number input)

B24. Out of the anal sex you had with casual male sex partners in the last month, how many times was a condom used? ___________(Number input)

B25. Of your casual male sex partners, how many partners were offered money or drugs to have sex with you?

______ (number input)

B26. Of your casual male sex partners, how many partners offered money, gifts, or drugs to have sex with you?

______ (number input)

B27. What’s the HIV status of your most recent casual partner?

- HIV positive
- HIV negative
- No idea (skip to B29)
- I never had a casual partner (skip to B30)

B28. How did you know the HIV status of your most recent casual male partner?

- He told me some time ago / I had known for some time
- He told me (online or in person) before sex
- I knew it from his profile on the Internet
- He made it clear without actually telling me
- Someone else told me
- We were at an event where everyone was HIV negative
- We were at an event where everyone was HIV positive
- I guessed
- Other reason

B29. Please count the number of your casual male sexual partner(s) with different HIV statuses in your lifetime:

- HIV positive ___________(number input)
- HIV negative ___________(number input)
- HIV unknown __________(number input)

B30. Who was your partner in your last sexual intercourse experience?

- Stable partner (Including boyfriend and regular sexual partner)
- Casual partner (Including irregular sexual partner and commercial sexual partner)

**C. Heterosexual Sex Situation (Q)**

*The next set of questions will ask about your sexual behaviors with females.*

C1. Have you ever had vaginal or anal sex with a female partner?

- Yes
- No (Skip to Section D)

C2. In the last three months, did you have any vaginal and/or anal sex with a female partner?

- Yes
- No (Skip to C11)

C3. In the last three months, how many females have you had vaginal or anal sex with?

_________female sex partners *(Number input)*

C4. In the last three months, when you had vaginal or anal sex with your female sex partner(s), how frequently did you use condoms?

- 0% condom use
- Less than 50% condom use
- More than 50% condom use
- 100% condom use

C5. In the last month, how many times have you had anal or vaginal sex with these women (combining all female sex partners)?

______ Sexual encounters (Number input)

C6. Out of the vaginal and/or anal sex you had with women in the past month, how many times was a condom used?

______Sex encounters (Number input)

C7. In the last three months, have you had sex with a stable female partner (who maintains a sexual relationship with you more than 3 months, such as a girlfriend, wife, or other regular female partner)?

- Yes
- No (Skip to C11)

C8. In the last three months, how many times have you had vaginal or anal sex with your stable female partner(s)?

_________sex encounters

C9. Out of the vaginal or anal sex you had with your stable female partner in the past three months, how many times was a condom used?

______sex encounters

C10. During you last sexual encounter with your stable female partner, in the past three months, did you use condoms?

- Yes
- No

C11. When you most recently had sex with a stable female partner, did you know about her HIV status?

- Yes, it’s positive
- Yes, it’s negative
- No (Skip to C13)
- I never had a stable female partner (Skip to C14)

C12. How did you know her HIV status?

- She told me some time ago / I had known for some time
- She told me (online or in person) before sex
- I knew it from her profile on the Internet
- She made it clear without actually telling me
- Someone else told me
- We were at an event where everyone was HIV negative
- We were at an event where everyone was HIV positive
- I guessed
- Other reason

C13. Please count the number of your stable female sex partner(s) with different HIV statuses in your lifetime:

- HIV positive ___________(number input)
- HIV negative ___________(number input)
- HIV unknown __________(number input)

C14. In the last three months, have you had sex with a casual female partner? (*Casual female partners mean female sex partners who maintain a sexual relationship with you equal to or less than 3 months, including commercial sex partners)*

- Yes
- No (Skip to C18)

C15. In the last three months, how many times have you had vaginal or anal sex with your casual female partners?

_________sex encounters

C16. Out of the vaginal or anal sex you had with your casual female partners in the past three months, how many times was a condom used?

______sex encounters

C17. During you last sexual encounter with your casual female partner, in the past three months, did you use condoms?

- Yes
- No

C18. When you had sex with your last casual or commercial female partner, did you know about her HIV status?

- Yes, it’s positive
- Yes, it’s negative
- No idea (Skip to C20)
- I never had a casual female partner (Skip to C21)

C19. How did you know her HIV status?

- She told me some time ago / I had known for some time
- She told me (online or in person) before sex
- I knew it from her profile on the Internet
- She made it clear without actually telling me
- Someone else told me
- We were at an event where everyone was HIV negative
- We were at an event where everyone was HIV positive
- I guessed
- Other reason

C20. Please count the number of your casual female partner(s) with different HIV statuses in your lifetime:

- HIV positive ___________(number input)
- HIV negative ___________(number input)
- HIV unknown __________(number input)

C21. Who was your partner in your last sexual intercourse experience with a female?

- Stable partner (including your wife, girlfriend, and regular female sexual partner)
- Casual partner (including irregular female sexual partner and commercial partner)

**D. HIV Testing Behavior (Q)**

D1. Have you ever been tested for HIV (including both facility-based and self-testing)?

- Yes
- No (Skip to F1)

D2. Have you been tested for HIV in the past 3 months?

- Yes (Would not send follow-up survey)
- No

D3. Where did you take the HIV testing during your last test?

- CDC (Skip to D5)
- Hospital (Skip to D5)
- Community organization (Skip to D5)
- The site for blood donation (Skip to D5)
- Last test was a self-test

*The next set of questions will ask about your practices and attitudes in regards to HIV testing (This is facility-based HIV testing, NOT HIV self testing).*

D4. Have you ever been tested for HIV (facility-based testing)?

- Yes
- No (Skip to E1)

D5. In the past year, how many times have you had a HIV facility-based testing?

_____________________(Number input)

D6. Of these HIV testings in the past year, how many times have you received the results? (Either positive or negative would count)

___________________(Number input)

D7. Did you receive post-test counseling?

- Yes
- No

D8. What was the result of your most recent HIV test?

- HIV positive/infected
- HIV negative/uninfected (skip to D14)
- I never got my test results (skip to D13)

D9. Did you receive a confirmatory Western Blot test?

- Yes
- No

D10. Are you currently taking any antiretroviral medicines?

- Yes
- No (Skip to D12)

D11. When did you start taking antiretroviral medicines?

_______________(date input)

D12. Did you used to be on treatment?

- Yes (please input the treatment duration from ___________to_____________) (skip to D14)
- No, I have never been on treatment (skip to D14)

D13. Have you ever been diagnosed with HIV?

- Yes
- No

D14. Did you notify your most recent stable male partner about your most recent HIV test result?

- Yes
- No
- I do not have a stable male partner (Skip to D17)

D15. Has your stable male partner ever been tested for HIV?

- Yes
- No （Skip to D17）
- I don't know （Skip to D17）

D16. What was the result of your stable male partner’s most recent HIV test?

- HIV positive/infected
- HIV negative/uninfected
- Never got test results
- I don’t know

D17. Who was with you when you most recently tested for HIV? (Select all that apply)

- No one, I was alone
- Partner
- Friend who is HIV negative or unknown
- Friend who is HIV positive
- Parent/family

D18. Did someone else (partner, boss, friend, or others) force you to take an HIV test (facility-based test)?

- Yes
- No

D19. What happened to your HIV testing frequency after your first HIV test?

- Increased
- Decreased
- No change

D20. What was the result of your first facility-based HIV testing?

- HIV positive/infected
- HIV negative/uninfected
- I never got my test result

D21. Has HIV testing (facility-based test) led to a violent confrontation (physical assault)?

- Yes
- No

**E. HIV Self-Testing (Q)**

*The next set of questions will ask about your HIV and STI testing and results. Self-testing refers to you administering the test yourself and interpreting results.*

E1. Have you ever taken an HIV self-test?

- Yes
- No (skip to F1)

E2. In the past year, how many times have you had a HIV self testing?

_____________________(Number input)

E3. Of these HIV testings in the past year, how many times have you received the results? (Either positive or negative would count)

___________________(Number input)

E4. Have you ever been administered (given) an HIV self-test from other people?

- Yes
- No

E5. Did someone else (partner, boss, friend, or other) force you to take an HIV self-test?

- Yes
- No

E6. Who was with you when you last took an HIV self-test? (Select all that apply)

- No one, I was alone
- Partner
- Friend who is HIV negative or unknown
- Friend who is HIV positive
- Parent/family

E7. Was your HIV self-test the first time you ever tested for HIV?

- Yes
- No

E8. What happened to your HIV testing frequency after your first HIV self-test?

- Increased
- Decreased
- No change

E9. Has your HIV self-test led to a violent confrontation (physical assault)?

- Yes
- No

E10. Did you have a positive result in any of your HIV self-test(s)?

- Yes
- No (skip to E12)

E11. Did you confirm your positive HIV self-test result at the CDC or hospital?

- Yes
- No

E12. Did you receive post self-test counseling?

- Yes
- No

**F. Testing Norms and Self-Efficacy**

*The following 12 questions will ask about your attitudes toward HIV testing. They ask for your honest opinions, so there are no right or wrong answers.*

F1. Most gay men who want to get tested but are afraid to get tested.

- Strongly agree
- Agree
- Disagree
- Strongly disagree

F2. Most gay men who get tested do not want others to find out they were tested.

- Strongly agree
- Agree
- Disagree
- Strongly disagree

F3. Most gay men want to get tested for HIV.

- Strongly agree
- Agree
- Disagree
- Strongly disagree

F4. Most gay men who want to get tested will tell their partners they want to get tested.

- Strongly agree
- Agree
- Disagree
- Strongly disagree

F5. Most gay men have been tested for HIV.

- Strongly agree
- Agree
- Disagree
- Strongly disagree

F6. Most gay men get tested for HIV only if they are sick or feel uncomfortable.

- Strongly agree
- Agree
- Disagree
- Strongly disagree

F7. You would feel comfortable discussing HIV testing with a potential partner.

- Strongly agree
- Agree
- Disagree
- Strongly disagree

F8. You feel confident that you could refuse to have sex with a partner who did not want to undergo HIV testing.

- Strongly agree
- Agree
- Disagree
- Strongly disagree

F9. You feel confident that you could persuade your partner to undergo HIV testing.

- Strongly agree
- Agree
- Disagree
- Strongly disagree

F10. You can have an HIV test if you wish.

- Strongly agree
- Agree
- Disagree
- Strongly disagree

F11. You will have an HIV test even if you are afraid to know the results.

- Strongly agree
- Agree
- Disagree
- Strongly disagree

F12. You have confidence that you will undergo HIV testing regularly.

- Strongly agree
- Agree
- Disagree
- Strongly disagree

F13. In the last twelve months, which of the following services did you receive:

- Condom distribution
- Lubricant distribution
- Peer Education
- STD Diagnosis or Treatment
- HIV counseling or Testing
- AIDS/STD Materials (pamphlets, etc.)
- Medical treatment (methadone, ART, etc.)
- Needle exchange
- None of the above

**G. Syphilis Testing**

G1. Have you ever been tested for syphilis?

- Yes
- No (Skip to Section H)

G2. Have you been tested for syphilis in the past 3 months?

- Yes
- No

G3. Has a doctor ever told you that you had syphilis?

- Yes
- No

**H. Community Engagement**

The following questionnaire is designed to measure the level of your engagement with the MSM community (i.e., tongzhi circle, gay online networks or groups) and at what level you are empowered. It is not a test, so there are no right or wrong answers.

H1. How important to you is community engagement and participation in developing HIV testing promotion campaigns (for your own community)?

- Very important
- Important
- Not that important
- Not important

H2. Are you aware of any ongoing community events promoting HIV testing among MSM?

- Yes
- No

H3. Have you ever volunteered at a health clinic or other location that provided sexual health services among MSM?

- Yes
- No

H4. Have you ever helped organize a testing and/or awareness campaign (e.g. HIV, condom use, etc.) that promoted sexual health among MSM?

- Yes
- No

H5. Have you ever encouraged someone else to get tested for HIV and/or another sexually transmitted disease?

- Yes
- No

H6. Have you ever accompanied a friend or partner to a testing facility to get tested for HIV and/or another sexually transmitted disease?

- Yes
- No

H7. Have you ever participated in online forums or discussions on social media (ie. Weixin, Weibo, Twitter, or other on-line communities) about HIV/STD testing or related services?

- Yes
- No

**I. HIV Testing Campaign Engagement**

*The next set of questions will ask you about your experiences with SESH’s creative contributory contest on HIV testing.*

I1. Have you heard about the SESH campaign or seen the advertisement for the campaign?

- Yes
- No (Skip to end of the section)

I2. Have you shared information regarding SESH with others on social media (WeChat, Weibo, etc.)?

- Yes
- No

I3. Have you participated in SESH’s in-person events? (Seminars, community events, designathon, receiving flyers, etc.)

- Yes
- No

I4. Have you submitted an entry (or entries) to the SESH HIV testing contest?

- Yes
- No

I5. Do you intend to help with contest organization or promotion in the future?

- Yes
- No

**J. Social media**

*This section asks you about your experiences with activities related to HIV testing online or using social media (Wechat, Weibo, QQ, mobile Apps, etc.).*

J1. Have you visited SESH’s WeChat official account (SESHglobal) ？

- Yes
- No

J2. Have you ever looked online for information about HIV testing?

- Yes
- No (Skip to J6)

J3. How frequently did you look online for information about HIV testing?

- Once a year at most
- Once every six months
- Once every three months
- Monthly
- Once a week at least

*Please try to recall the LAST time you searched online for information on HIV testing:*

J4. Did you intend to: (Select all the choices that apply to you)

- Search about the process involved with HIV testing
- Find where to receive an HIV test
- Get more information and knowledge about HIV testing
- Talk to other people about HIV testing
- Others

J5. Have you used the following platforms to start searching? (**Select all the choices that apply to you**)

- At a search engine (like Baidu, Sougou)
- At a site that specializes in HIV testing information (like Gztz, Danlan)_
- At a more general Q&A site that contains information on all kinds of topics (like Baidu Zhidao, Aiwen, Zhihu)
- At a social network site (like Wechat, Weibo, QQ)
- At a mobile App (like Blued, Jackd, Grindr)
- Other________

J6. Have you ever seen anything related to HIV testing on Weibo, Wechat, QQ messages or mobile Apps?

- Yes
- No (Skip to K1)

J7. **In the last three months**, have you seen anything related to HIV testing on Weibo, Wechat, QQ messages or mobile Apps?

- Yes
- No (Skip to K1)

J8. Please specify if you have conducted the following behaviors on any of the listed social media platforms in the last 3 months? (Select all that apply)

| Behaviors | Social Media Platforms | | | | |
| --- | --- | --- | --- | --- | --- |
| Received information about HIV testing | Weibo | Wechat | QQ | App | None |
| Liked information about HIV testing | Weibo | Wechat | QQ | App | None |
| Forwarded information about HIV testing | Weibo | Wechat | QQ | App | None |
| Followed subscription account(s) about HIV testing | Weibo | Wechat | QQ | App | None |
| Shared information about HIV testing | Weibo | Wechat | QQ | App | None |

| Behaviors | Social Media Platforms | | | | |
| --- | --- | --- | --- | --- | --- |
| Posted information about HIV testing | Weibo | Wechat | QQ | App | None |
| Commented about HIV testing | Weibo | Wechat | QQ | App | None |
| Privately discussed about HIV testing | Weibo | Wechat | QQ | App | None |
| Discussed in groups about HIV testing | Weibo | Wechat | QQ | App | None |

**K. Anticipated HIV Stigma**

*Please consider the following statements and indicate to what extent you agree or disagree with them.*

K1. If I had HIV, I’d worry about people discriminating against me.

- Strongly Agree
- Agree
- Disagree
- Strongly Disagree

K2. If I got infected with HIV no one would date or become involved with me.

- Strongly Agree
- Agree
- Disagree
- Strongly Disagree

K3. If I got infected with HIV, no one would want to have sex with me.

- Strongly Agree
- Agree
- Disagree
- Strongly Disagree

K4. If I got infected with HIV, I would work hard to keep my HIV status a secret.

- Strongly Agree
- Agree
- Disagree
- Strongly Disagree

K5. Upon learning I contracted HIV, I would feel set apart and isolated from the rest of the world.

- Strongly Agree
- Agree
- Disagree
- Strongly Disagree

K6. If I got infected with HIV, I would feel I was not as good a person as others.

- Strongly Agree
- Agree
- Disagree
- Strongly Disagree

K7. I would never feel ashamed of getting HIV.

- Strongly Agree
- Agree
- Disagree
- Strongly Disagree

**Intervention page [ONLY TO GZ/YT]**

**Thank you very much! You have finished all the questions now. Please view this picture for 15s, and then you can fill out your information for receiving the rewards.**

**[Poster 1]**

**[Slogan 1]**

**[Locations]**

**In the next 3 months, we will send you images and texts relevant to the HIV testing in every 2 weeks.**

**Ending section**

R1. Would you like to receive the reward as a wechat red pocket or mobile phone top-up?

- Wechat red pocket: _______ (wechat account) (Skip to R3)
- Mobile phone top-up (Skip to R2)

(Appear if choose “Wechat red pocket”) Please add our wechat named “SESHSurvey” to get reward. Please be assured that all of your personal information will be protected by a password secured phone. Only staff who would send out the reminder and reward could see your account. We will not share your personal information to anyone, and will not contact you except for the situation of follow up notifications and reward deliveries. After we finish all the follow up surveys, your contact will be deleted.

(To those who did HIV testing in the past 3 months or are currently living with HIV, jump to the end directly)

R2. Thanks! You have finished our survey. In the following year, you will receive up to 4 follow up surveys (one about every three months). Each time you complete a survey, you will receive 50 RMB. If you complete all four surveys, you will have a chance to win the latest iPad mini. To deliver our follow-up notifications, we hope that we can add you as our survey wechat friend.

Please be assured that all of your personal information will be protected by a password secured phone.Only staff who would send out the reminder and reward could see your account. We will not share your personal information to anyone, and will not contact you except for the situation of follow up notifications and reward deliveries. After we finish all the follow up surveys, your contact will be deleted.

Please add our wechat account.

- Yes: ______ (Wechat number input)
- No
- I do not have a wechat account

R3. Thanks! You have finished our survey. In the following year, you will receive up to 4 follow up surveys (one about every three months). Each time you complete a survey, you will receive 50 RMB. If you complete all four surveys, you will have a chance to win the latest iPad mini.
