# Supplementary material for: Crowdsourcing to promote HIV testing among MSM in China: study protocol for a stepped wedge randomized controlled trial
Source: Trials. 2017 Oct 2;18:447. doi: 10.1186/s13063-017-2183-1 (PMC5625620; doi:10.1186/s13063-017-2183-1)
Supplement: Supplementary file 2 — CDC surveillance survey instrument (English version). This is the English version of the CDC surveillance survey baseline online used at CDC surveillance sites. (DOCX 46 kb) [file 13063_2017_2183_MOESM2_ESM.docx]

**Additional File 2. CDC surveillance survey instrument**

CDC Surveillance Survey Questions

1. Excluding this visit to the CDC, in the past year, how often have you received an HIV test?
2. Never
3. Once
4. about twice per year or more frequent
5. Did not receive an HIV test in the past year, but have received HIV test before

1. Excluding this visit to the CDC, in the past year, how often have you received a syphilis test?
2. Never
3. Once
4. about twice per year or more frequent
5. Did not receive an HIV test in the past year, but have received HIV test before
6. In the past three months, have you seen any videos to promote HIV testing?
   1. Yes

b) No

4) Have you had an HIV Test in the past three months?

- 1. Yes
  2. No

5) Have you ever conducted an HIV Self-Testing in the last two years? [HIV self-testing refers to you administering the test yourself and interpreting results]

- 1. Once every two years
  2. Once a year
  3. Once every six months
  4. Once every three months
  5. Monthly
  6. Never

**Engagement in Health Promotion Campaigns**

6) Are you aware of any ongoing community events promoting HIV testing among MSM?

1. Yes
2. No (Skip to question 8)
3. Have you ever helped organize a testing and/or awareness campaign that promoted HIV testing among MSM?
   1. Yes
   2. No
4. Have you ever volunteered at a health clinic or other location that provided HIV testing services among MSM?
   1. Yes
   2. No
5. Have you ever encouraged someone else to get tested for HIV?
   1. Yes
   2. No
6. Have you ever accompanied a friend or partner to a testing facility to get tested for HIV? [Testing facilities refers to any CDC authorized testing facilities]
   1. Yes
   2. No
7. Have you ever looked online for information about HIV testing?
8. Yes
9. No (Skip to 14)
10. Within the past two years, how frequently did you look online for information about HIV testing?
11. Once every two years
12. Once a year
13. Once every six months
14. Once every three months
15. Monthly
16. Weekly
17. Daily
18. When you looked for HIV testing information online, have you used the following platforms? (**Select all that apply**)
19. Search engine such as Baidu, haosou, sougou
20. Sites that specializes in HIV testing information, like GZTZ, danlan, Shandongtongzhi
21. More general sites like baidu zhidao, aiwen, zhihu, that contains information on all kinds of topics,
22. Social network platforms such as wechat, weibo, QQ
23. Mobile Apps such as Blued, Jack’d, Grindr
24. Other________
25. In the last three months, have you received, liked, posted, followed, discussed, shared, commented anything related to HIV testing on Weibo, Wechat, QQ messages or mobile Apps?
26. Yes
27. No (Skip to question 16)
28. Please specify whether you have conducted the following behaviors on Weibo, Wechat, QQ messages or mobile Apps:

| Received information about HIV testing | - Yes | - No |
| --- | --- | --- |
| Liked information about HIV testing | - Yes | - No |
| Forwarded information about HIV testing | - Yes | - No |
| Followed subscription account about HIV testing | - Yes | - No |
| Posted information about HIV testing | - Yes | - No |
| Shared information about HIV testing | - Yes | - No |
| Commented about HIV testing | - Yes | - No |
| Privately discussed about HIV testing | - Yes | - No |
| Discussed in groups about HIV testing | - Yes | - No |

**Condom Use Questions**

1. In the last three months, did you have any condomless sex with any male sex partner (casual, main, or other)?
   1. Yes
   2. No (Skip to question 18)
2. In the last three months, did you have any condomless sex with any female sex partner (casual, main, or other)?
   1. Yes
   2. No

**If you are HIV positive, please answer the following questions:**

1. Have you received a WB/diagnostic test within 3 months of initial positive HIV test?
   1. Yes
   2. No (Skip question 19)
2. Did you go to conduct CD4 test within 30 days and 90 days respectively after the HIV diagnosis?
   1. Yes
   2. No
