# Supplementary material for: Crowdsourcing to promote HIV testing among MSM in China: study protocol for a stepped wedge randomized controlled trial
Source: Trials. 2017 Oct 2;18:447. doi: 10.1186/s13063-017-2183-1 (PMC5625620; doi:10.1186/s13063-017-2183-1)
Supplement: Supplementary file 3 — Table for secondary outcomes. This table lists the secondary outcomes measured in this RCT and their definitions. (DOCX 35 kb) [file 13063_2017_2183_MOESM3_ESM.docx]

**Additional File 3. Table for secondary outcomes**

| **Secondary Outcome** | **Definition** |
| --- | --- |
| *Incremental cost (overall)* | Incremental cost, defined as the cost associated with respective interventions (development, start-up, implementation, condom use, intervention per individual who reported no sex or sex with a condom during the follow-up period.) |
| *Incremental cost for HIV self-testing* | Incremental cost for HIV self-testing, defined as the cost associated with HIV self-testing (development, start-up, implementation, condom use, intervention per individual who reported no sex or sex with a condom during the follow-up period.) |
| *Condom use* | Frequency of men, defined as the number of men who reported increased condom use (in anal, vaginal, and/or oral sex with either male and female sex partners) comparing pre-intervention and post-intervention values |
| *HIV testing social norms* ^1^ | Frequency of men, defined as number of men who report higher levels of social norms when comparing their pre-intervention and post-intervention HIV testing norms |
| *HIV testing self-efficacy* ^2^ | Frequency of men, defined as number of men who had an increase in HIV testing self-efficacy when comparing their pre-intervention and post-intervention self-efficacy |
| *Community engagement/ MSM community affiliation* | Frequency of men, defined as an increase in closer affiliation with the MSM community (i.e., tongzhi circle, gay online networks or groups) when comparing their pre-intervention and post-intervention engagement |
| *Campaign engagement* | Frequency of men, defined as number of men who had an increase in taking part in the HIV testing campaign when comparing their pre-intervention and post-intervention engagement |
| *HIV self-testing (self-report)* | Frequency of men, defined as the number of men who reported being self-tested for HIV during the previous three months |
| *HIV self-testing (confirmed)* | Frequency of men, defined as the number of men who received HIV self-test kits during the previous three months |
| *Anticipated HIV stigma* ^3^ | Frequency of men, defined as number of men who report anticipated HIV stigma when comparing their pre-intervention and post-intervention |
| *Syphilis testing* | Frequency of men, defined as the number of men who reported being tested for syphilis (excluding HIV) during the previous three months |
| *Weibo engagement* | Frequency of men, defined as the number of men who reported using Weibo in the past three months to give or receive information about HIV testing comparing their pre-intervention and post-intervention engagement (Except the intervention delivered by SESH) |
| *Wechat engagement* | Frequency of men, defined as the number of men who reported using Wechat in the past three months to give or receive information about HIV testing comparing their pre-intervention and post-intervention engagement (Except the intervention delivered by SESH) |
| *QQ engagement* | Frequency of men, defined as the number of men who reported using QQ in the past three months to give or receive information about HIV testing comparing their pre-intervention and post-intervention engagement (Except the intervention delivered by SESH) |
| *Mobile app engagement* | Frequency of men, defined as the number of men who reported using mobile apps in the past three months to give or receive information about HIV testing comparing their pre-intervention and post-intervention engagement |

^1^ HIV testing social norms will be measured using six survey items that are each on a five point Likert scale. Increased HIV testing social norms will be defined as having an increase from baseline in any two of these six survey items and dichotomized accordingly (social norm change will be categorized into three groups: increased, stable and decreased social norm). The HIV testing social norm outcome will be assessed in the entire group as well as the subgroup of men who were referred by their friends. This is adapted from Pettifor, A., MacPhail, C., Suchindran, S, & Delany-Moretlwe, S. (2015). Factors associated with HIV testing among public sector clinic attendees in Johanesburg, South Africa. AIDS and Behavior, 14, 913-921.

^2^ Self-efficacy will be measured using six survey items that are each on a five point Likert scale. Increased self-efficacy will be defined as having an increase from baseline in any two of these seven survey items and dichotomized accordingly (self-efficacy change will be categorized into three groups: increased, stable and decreased self-efficacy). This is adapted from Gu, J., Lau, J. T. F., & Tsui, H. (2011). Psychological factors in association with uptake of voluntary counselling and testing for HIV among men who have sex with men in Hong Kong. Public Health, 125, 275-282.

^3^ The Anticipated HIV Stigma will be assessed using 7 survey items that are each on a four point Likert scale. Reduced anticipated HIV stigma will be defined as having a decrease from baseline in the mean score (Continuous variable). This is adapted from Golub, S. A. & Gamarel, K. E. (2013). The impact of anticipated HIV stigma on delays in HIV testing behaviors: Findings from a community-based sample of men who have sex with men and transgender women in New York City. AIDS Patient Care and STDs, 27(11), 621-627.
