# Supplementary material for: Crowdsourcing to promote HIV testing among MSM in China: study protocol for a stepped wedge randomized controlled trial
Source: Trials. 2017 Oct 2;18:447. doi: 10.1186/s13063-017-2183-1 (PMC5625620; doi:10.1186/s13063-017-2183-1)
Supplement: Supplementary file 4 — Table for sample size calculation. This table lists the variables and values involved in the sample size calculation. (DOCX 46 kb) [file 13063_2017_2183_MOESM4_ESM.docx]

**Additional File 4. Table for sample size calculation**

| **P_i_*** | **P_c_^$^** | **Number of clusters** | **Number of time periods** | **Coefficient of variation^@^** | **Alpha** | **Power** | **Sample size/no LTFU** | **Loss to follow up** | **Sample size for each cluster** | **Total sample size (8 clusters)** |
| --- | --- | --- | --- | --- | --- | --- | --- | --- | --- | --- |
| 30 | 20 | 8 | 5 | 0.4 | 0.05 | 0.9 | 80 | 30% | 114 | 912 |
| 30 | 20 | 8 | 5 | 0.15 | 0.05 | 0.9 | 71 | 30% | 101 | 808 |
| 30 | 15 | 8 | 5 | 0.4 | 0.05 | 0.9 | 31 | 30% | 44 | 352 |
| 30 | 15 | 8 | 5 | 0.15 | 0.05 | 0.9 | 26 | 30% | 37 | 296 |
| **35** | **25** | **8** | 5 | **0.4** | **0.05** | **0.9** | **91** | **30%** | 130 | **1040** |
| 35 | 25 | 8 | 5 | 0.15 | 0.05 | 0.9 | 83 | 30% | 119 | 952 |
| 35 | 20 | 8 | 5 | 0.4 | 0.05 | 0.9 | 37 | 30% | 53 | 424 |
| 35 | 20 | 8 | 5 | 0.15 | 0.05 | 0.9 | 31 | 30% | 44 | 352 |
| 40 | 30 | 8 | 5 | 0.4 | 0.05 | 0.9 | 99 | 30% | 141 | 1128 |
| 40 | 30 | 8 | 5 | 0.15 | 0.05 | 0.9 | 93 | 30% | 133 | 1064 |
| 40 | 25 | 8 | 5 | 0.4 | 0.05 | 0.9 | 42 | 30% | 60 | 480 |
| 40 | 25 | 8 | 5 | 0.15 | 0.05 | 0.9 | 36 | 30% | 51 | 408 |
| 45 | 35 | 8 | 5 | 0.4 | 0.05 | 0.9 | 104 | 30% | 149 | 1192 |
| 45 | 35 | 8 | 5 | 0.15 | 0.05 | 0.9 | 99 | 30% | 141 | 1128 |
| 45 | 30 | 8 | 5 | 0.4 | 0.05 | 0.9 | 45 | 30% | 64 | 512 |
| 45 | 30 | 8 | 5 | 0.15 | 0.05 | 0.9 | 40 | 30% | 57 | 456 |

Note: *Pi: probability of HIV testing during intervention period; ^$^Pc: probability of HIV testing during control period; ^@^ usually between 0.15-0.4.
